# A Biogeography-Based Optimization Algorithm Hybridized with Tabu Search for the Quadratic Assignment Problem

**DOI:** 10.1155/2016/5803893

**Published:** 2015-12-27

**Authors:** Wee Loon Lim, Antoni Wibowo, Mohammad Ishak Desa, Habibollah Haron

**Affiliations:** ^1^Faculty of Computing, Universiti Teknologi Malaysia (UTM), 81310 Johor Bahru, Johor, Malaysia; ^2^School of Quantitative Sciences, UUM College of Arts and Sciences, Universiti Utara Malaysia, 06010 Sintok, Kedah, Malaysia; ^3^Faculty of Information and Communication Technology, Universiti Teknikal Malaysia Melaka, Hang Tuah Jaya, 76100 Durian Tunggal, Melaka, Malaysia

## Abstract

The quadratic assignment problem (QAP) is an NP-hard combinatorial optimization problem with a wide variety of applications. Biogeography-based optimization (BBO), a relatively new optimization technique based on the biogeography concept, uses the idea of migration strategy of species to derive algorithm for solving optimization problems. It has been shown that BBO provides performance on a par with other optimization methods. A classical BBO algorithm employs the mutation operator as its diversification strategy. However, this process will often ruin the quality of solutions in QAP. In this paper, we propose a hybrid technique to overcome the weakness of classical BBO algorithm to solve QAP, by replacing the mutation operator with a tabu search procedure. Our experiments using the benchmark instances from QAPLIB show that the proposed hybrid method is able to find good solutions for them within reasonable computational times. Out of 61 benchmark instances tested, the proposed method is able to obtain the best known solutions for 57 of them.

## 1. Introduction

The quadratic assignment problem (QAP) belongs to the class of combinatorial optimization. This problem concerns assigning a number of facilities to the same number of locations, with the objective to find a way of assignment such that the total cost involved is minimized. The total cost of the QAP is the product of flow and distances between the facilities.

Sahni and Gonzalez [1] have shown that QAP is NP-hard. Therefore, unless P = NP, it is impossible to find optimal solutions in polynomial time. The complexity of QAP draws the interest of researchers worldwide over the past few decades. More than 300 papers have been published for the theory, applications, and solution techniques for the QAP [2]. Despite the extensive research done, QAP remains one of the most difficult combinatorial optimization problems. Generally, QAP instances with problem size > 30 cannot be solved within reasonable computational times. In the literature, a lot of heuristic approaches to the QAP have been proposed. Some of the common methods are simulated annealing, tabu search (TS), genetic algorithm (GA), and ant colony optimization [3–10].

Among the exact methods for QAP, branch and bound (BB) algorithm is the most efficient [11, 12]. Currently, the largest instance solved by BB algorithm is of size 36 [4]. Solving QAP instances of size 20 was impossible before 1990s. It was until 1994 when Mautor and Roucairol [13] gave the exact solutions for nug16 and els19. The BB algorithm of Brixius and Anstreicher [14] solved the nug25 instance after 13 days of CPU time using sequential processing. The kra30a was solved by Hahn and Krarup [15] after 99 days of work with a sequential workstation. Nystrom [16] provided the exact solutions for the ste36b and ste36c after 200 days of work using a distributed environment. The nug30 remained unsolved until 2002 when Anstreicher et al. [17] reported the exact solution with seven days of work using 650 processors. Basically, the exact methods require much more computational resources and time than the heuristic algorithms. This is the reason we did not use exact methods in this study.

The QAP has a wide variety in the real-world applications with facility layout problem being the most popular applied area. These include campus planning, backboard wiring, hospital planning, zoning in a forest, and the placement of electronic components. Besides, the QAP is also used for typewriter keyboard design, development of control boards, turbine runner problem, ranking of archaeology data, scheduling of production lines analysis of chemical reactions, data analysis, economic problems, VLSI circuit and system design, website structure, and optimizing of Arabic keyboard design. Comprehensive review of QAP can be referred to in [2, 18].

Biogeography is a study of the geographical distribution of biological organisms. The science of biogeography can be referred to in the work of two naturalists in the nineteenth century named Wallace [19] and Darwin [20]. In the 1960s, MacArthur and Wilson [21] started to work on mathematical models of biogeography. Their study focused on the distribution of species among neighboring habitats and how species migrate from one habitat to another. However, it was until 2008 when Simon [22] presented a new optimization method based on it, namely, biogeography-based optimization (BBO). In his work, Simon applied BBO to the problem of sensor selection for aircraft engine health estimation and the performance of BBO is at a par with other population-based methods such as ant colony optimization, differential evolution, GA, and particle swarm optimization (PSO). The good performance of BBO on the sensor selection problem proves that it can be successfully applied to practical problems.

In his next paper, Simon [23] showed that BBO outperforms GA when both of them have a low mutation rate. Furthermore, BBO has shown a good performance when being applied to real-world optimization problems such as classification of satellite images, groundwater detection, and the solving of complex economic load dispatch [24–26].

The good performance of BBO on complex optimization problems inspired us to apply this iterative and population-based method to QAP. A classical BBO algorithm uses migration and mutation operators as its intensification and diversification strategy, respectively. During the iterated process of BBO algorithm, migration operator tends to improve every solution within the population while mutation operator increases the diversity among them. However, quite often the mutation operator will ruin the quality of solutions.

In order to overcome the weakness of mutation operator, we propose to replace it with a tabu search procedure. TS is a heuristic procedure for solving the optimization problems. A typical TS starts with defining a neighborhood, in which a given solution may move to and produce a new one. TS will evaluate each of the neighboring solutions and choose the move that improves the objective function the most. If there is no improving move, TS will choose one that weakens the objective function the least. The main difference of TS from other local search techniques is that it will use memory to store the recent moves. These moves are then forbidden and thus avoiding the search procedure to return to the local optimum just visited.

The characteristics of TS described above make it a suitable replacement for the mutation operator of classical BBO. Not only the diversity of the population is maintained but at the same time it prevents the quality of solutions within the population from being ruined. We choose to incorporate the robust tabu search (RTS) of Taillard [27] into our BBO algorithm for QAP because it is efficient and requires fewer parameters in its implementation.

In this paper, we propose a BBO algorithm hybridized with tabu search (BBOTS) for QAP. We apply it to the benchmark instances obtained from QAPLIB [28] with size ranging from 12 to 80. The experimental results demonstrate that the proposed algorithm outperforms the classical BBO algorithm with mutation operator. Our hybrid algorithm is able to solve most of the benchmark instances of QAP tested.

## 2. Problem Definition

The QAP is an assignment model in which a set of facilities is supposed to be allocated to a set of locations, with each location having exactly one facility. The objective is to find a way of assignment such that the total cost involved is minimized. The total cost is calculated by multiplying the flow and the distances between facilities.

Let *n* be the number of facilities and locations, given two *n* × *n* matrices as follows:

 
*F* = (*f*_*ik*_), where *f*_*ik*_ is the flow from facility *i* to facility *k*;
 
*D* = (*d*_*jl*_), where *d*_*jl*_ is the distance from location *j* to location *l*.

The Koopmans-Beckmann [29] form of the QAP can be formulated as follows:

(1) $$\begin{array}{c} \underset{\varphi \in S_{n}}{\min}\left[\;\overset{n}{\underset{i=1\;}{\sum }}\overset{n}{\underset{\;k=1}{\sum }}f_{ik}d_{\varphi \left(i\right)\varphi \left(k\right)}\;\right], \end{array}$$

where *S*_*n*_ is the set of all permutations of the integers 1,2,…, *n*. Each product of *f*_*ik*_ *d*_*φ*(*i*)*φ*(*k*)_ is the cost for assigning facility *i* to location *φ*(*i*) and facility *k* to location *φ*(*k*). There are several ways in mathematics to represent an assignment. Throughout this paper, we use the representation of assignment by permutation.

## 3. Methods

In this section, we will first introduce BBO as general. This includes explaining the terminology used and its difference with another popular population-based optimization method, the GA. Next, we will explain the proposed algorithm in detail. We developed two BBO algorithms for QAP:

- a classical BBO algorithm with mutation operator,
- a BBO algorithm hybridized with tabu search.

Computational experiments were carried out to show the difference in performance between them. Note that the implementation of both algorithms is identical, except for the mutation step. Therefore, all the other steps of the algorithms are explained only once.

### 3.1. Biogeography-Based Optimization

BBO is a new algorithm inspired by biogeography, which studies the geographical distribution of biological organisms. MacArthur and Wilson [21] worked together on the mathematical models of biogeography in the 1960s. They focused primarily on the distribution of species among neighboring habitats and how species migrate from one habitat to another. Since then, biogeography has become a major area of research. However, it was until 2008 when Simon [22] generalized it to obtain a general-purpose optimization algorithm.

Just like other biology-based algorithms, for example, GA and PSO, BBO is a population-based algorithm in which a population of candidate solutions is used in the searching procedure for global optima. BBO has certain common features with another popular optimization algorithm, the GA. In GA, an individual within the population is called a chromosome and has its own fitness value. Likewise in BBO, each individual is termed as a habitat and has its habitat suitability index (HSI) to evaluate its quality as a solution. Since we are dealing with a minimization problem, a low-HSI habitat represents a good solution and a high-HSI habitat is a poor solution instead. Each chromosome in GA consists of genes, while, for BBO, each habitat is characterized by Suitability Index Variables (SIVs). There are two main operators in GA, which are crossover and mutation. Meanwhile, in BBO, the main operators are migration and mutation. The migration operator consists of emigration and immigration. It is used to improve and evolve the habitats (solutions to the optimization problem) in the population. Solution features (SIVs) emigrate from low-HSI habitats (emigrating habitats) to high-HSI habitats (immigrating habitats). In other words, high-HSI habitats accept new features from low-HSI habitats through the immigration process. There are different alternatives for migration and mutation process of BBO. The way we implement these two operators are explained in detail in the later part.  Table 1 compares the characteristics of BBO and GA.

The classical BBO algorithm proposed by Simon [22] can be described with the algorithm in the following.

*A Classical BBO Algorithm*

1. Initialize the BBO parameters (this includes deriving a representation scheme for habitats, which is problems dependent, and also initializing the maximum migration rate, maximum mutation rate, and elitism parameter).
2. Initialize a random set of habitats, corresponding to the potential solutions.
3. Associate each habitat with immigration and emigration rate based on their HSI.
4. Probabilistically perform migration to modify each one-elite habitat. Then, recompute each HSI.
5. Associate each habitat with mutation rate based on their species count.
6. Probabilistically perform mutation to modify each nonelite habitat. Then, recompute each HSI.
7. Go to step (3) for the next iteration. Repeat until a predefined number of generations are reached, or after an acceptable solution is found.

### 3.2. The Proposed Algorithm

#### 3.2.1. Representing Scheme of Habitats

As mention earlier, a habitat is represented by a permutation of integer 1,2,…, *n*, in which *n* is the number of facilities and locations, that is, the problem size. For example, (2, 5, 1, 4, 3) is a possible habitat for an instance of QAP with problem size of 5. In this case, “2” of (2, 5, 1, 4, 3) means facility 1 is placed at location 2, and it is a SIV of this habitat. Likewise, “5” means facility 2 is placed at location 5 and so on.

#### 3.2.2. Initialization of the BBO Algorithm

The BBO algorithm starts with a population of randomly generated habitats. In our algorithm, a habitat is represented by a permutation of integers. A permutation is randomly generated and will only be inserted if it does not exist in the population yet. This is to avoid having duplicate habitats in the initial population and hence enhancing the diversity.

#### 3.2.3. Selection Strategy for Migration

In BBO, a good habitat (solution) is one with low HSI. Good habitats tend to share their features with poor habitat. This is done by migrating SIVs from emigrating habitats to immigrating habitats. In order to perform migration, we will first use immigration rates (*λ*_*k*_) of a habitat to decide whether to modify it, then we use emigration rates (*μ*_*k*_) of other habitats to decide which of them should migrate a SIV to the first habitat.

According to the biogeography, the SIVs of a good habitat (with low HSI) tend to emigrate to a poor habitat (with high HSI). Therefore, a good habitat has relatively high *μ*_*k*_ and low *λ*_*k*_, while a poor solution has relatively low *μ*_*k*_ and high *λ*_*k*_.

The immigration rate *λ*_*k*_ and the emigration rates *μ*_*k*_ are calculated with the following equations, respectively,

(2) λk=I1−kh,

(3) μk=Ekh.

In the equations, *k* represents the rank of a habitat after sorting them in accordance to their HSI. Habitats with high HSI (a poor solution) have lower rank while habitats with low HSI will have higher rank. In other words, the habitats are sorted from the worst to the best. In the equations, *h* is the number of habitats in the population, while *I* is the maximum immigration rate and *E* the maximum emigration rate which are both usually set to 1.

**Figure 1:**
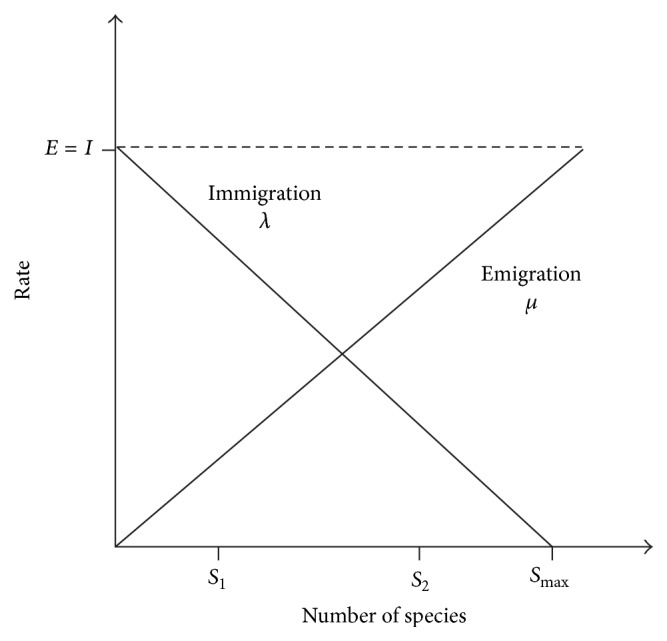
Model for immigration and emigration rates.

Figure 1 illustrates the model for immigration and emigration rates [22]. In the figure, *S*_1_ is a relatively poor solution, while *S*_2_ is a relatively good solution. Although in the figure the immigration and emigration rate are considered linear, they can actually be replaced with curves in which better performance might be attained. In fact, Mussetta and Pirinoli [30] have shown that a variation of BBO with quadratic migration model and restart procedure outperforms the classical BBO when being applied to several benchmark functions. The selection strategy is summarized with the algorithm in Algorithm 1.

#### 3.2.4. Selection Strategy for Mutation

In BBO, each habitat has an associated probability for them to exist as a solution to the given problem. The probability that whether mutation occurs in a habitat is called the mutation rate. To determine the mutation rate for each habitat, we must first evaluate the species count probability with the following equation:

(4) $$\begin{array}{c} P=\frac{v}{\sum_{i=1}^{h+1}v_{i}}, \end{array}$$

in which *v* and *v*_*i*_ are evaluated by

(5) v=v1v2⋯vh+1T,vi=h!h+1−i!i−1!i=1,2,…,i′vh+2−ii=i′+1,i′+2,…,h+1,

where *i*′ = ceil((*n* + 1)/2). The mutation rate *m*(*S*) is inversely proportional to the species count probability; therefore, we have

(6) $$\begin{array}{c} m\left(\;S\;\right)=m_{\max}\left(\;\frac{1-P_{s}}{P_{\max}}\;\right), \end{array}$$

where *m*_max⁡_ is the maximum mutation rate and *P*_max⁡_ the largest species count probability. The details on how to derive the above formulae can be referred to in [22].

#### 3.2.5. Migration Operator

As mentioned earlier, the SIVs from a good habitat tend to migrate into a poor habitat. This migration operator is performed probabilistically based on immigration and emigration rates. In this section, we will explain how the migration is implemented in our BBOTS algorithm.

Consider dealing with an instance of QAP with problem size of 5. Suppose, based on immigration and emigration rates, that an emigrating habitat, *H*_*e*_ = (2,4, 3,5, 1), and an immigrating habitat, *H*_*i*_ = (5,3, 1,4, 2), are selected. Therefore, a SIV of *H*_*e*_ will be randomly selected and replaces a randomly selected SIV of *H*_*i*_.

**Figure 2:**
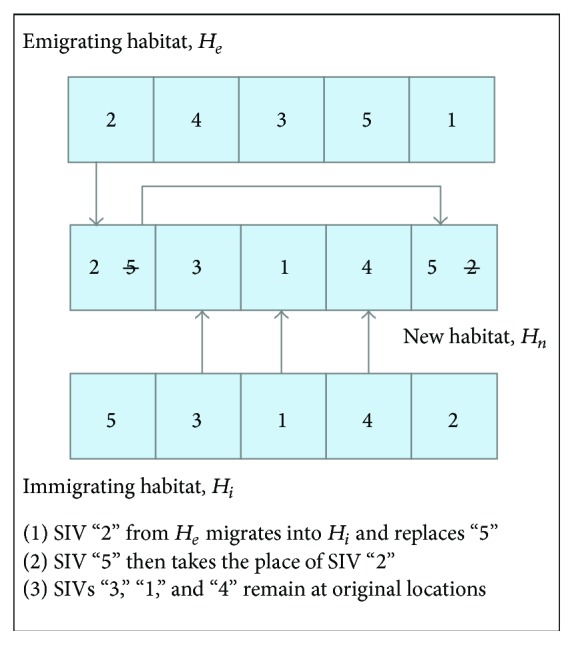
Migration operator of BBOTS algorithm.

Assuming the first element of *H*_*e*_, “2” is selected to replace the first SIV of *H*_*i*_, “5.” Therefore, the new habitat, *H*_*n*_ = (2,3, 1,4, 2), is produced. However, it is not a feasible solution for our problem. In order to maintain the feasibility of a solution, once a new SIV migrates into a habitat, the old SIV will replace the SIV with the same value of newly emigrating SIV. Therefore, the new habitat, *H*_*n*_, will be (2, 3, 1, 4, 5).  Figure 2 illustrates this step clearly.

Once a new habitat is produced, it will only be accepted into the population only if it is not the same with any existing habitat. This is to enhance the diversity throughout the population. Besides, we use the concept of elitism to prevent the best solutions from being corrupted by immigration. This is done by setting the immigration rate for best solutions to 0.

#### 3.2.6. Mutation Operator

According to biogeography, cataclysmic events may happen from time to time, drastically changing the characteristics of a natural habitat. In BBO algorithm, this is imitated through a mutation operator. This process is important to increase the diversity among population.

In classical BBO algorithm, a mutation is performed by simply replacing a selected SIV of a habitat with a randomly generated SIV. Remember, in BBO, migration operator serves as intensification strategy, while mutation operator is used to maintain the diversity. Habitats within the population are improved by migration operator throughout the iteration of BBO algorithm. However, this effort might be ruined by the mutation operator because the quality of the mutated habitats is not guaranteed. Quite often, the mutation process will result in a poor habitat.

A simple solution for this drawback comes in mind such that after performing the mutation, the mutated habitats are kept in the population only if the quality is better than the original habitats. However, this is not practical when solving a complex optimization problem such as QAP. Most of the time, the resulting habitats from a simple mutation operator are unlikely to be better than the original habitats, especially as the algorithm converges.

To overcome this weakness of classical BBO algorithm, we propose to replace the mutation operator with a tabu search procedure. Proposed by Glover [31], TS is a metaheuristic which performs local search based on the information in the memory. TS is both neighborhood-based and iterative. At each iteration, current solution will make a move to the neighborhood solution with the best objective function value. To avoid trapping in local optima, the move that has been made will be stored in a tabu list and a reverse move to previous solutions is forbidden. The performance of tabu search highly depends on the neighborhood type used and tabu list implementation.

The advantages of replacing the mutation operator of classical BBO algorithm with TS come in two points. First, the original aim of mutation process is maintained, which is to increase the diversity of the population. Besides, at the same time, the quality of the resulting habitats is prevented from being ruined.

In order to prove the above statement, we developed two BBO algorithms for QAP:

- a classical BBO algorithm with mutation operator,
- a BBO algorithm hybridized with tabu search.

**Figure 3:**
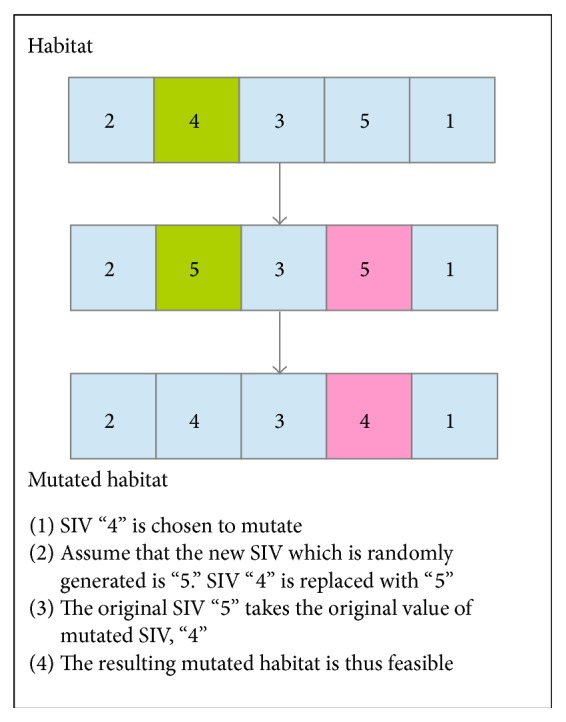
The mutation process of classical BBO algorithm for QAP.

For the classical BBO algorithm, the SIV of a habitat will be chosen to undergo mutation based on the mutation rate. The mutation process is then performed by replacing the old SIV with another randomly generated SIV. After that, SIV of the habitat, which is of the same value with the randomly generated SIV, will inherit the original value of the mutated SIV. Thus, the resulting habitat will remain a feasible solution.  Figure 3 illustrates the mutation process of the classical BBO algorithm for QAP.

For the proposed BBOTS algorithm, we chose to replace the mutation operator with robust tabu search (RTS) of Taillard [27]. Despite being developed long time ago (1991), RTS is still one of the best performing algorithms for QAP.

The tabu list of RTS consists of pairs of facilities that cannot be exchanged. In the tabu list, the latest iteration at which a pair of facilities is placed at certain locations is stored. A swap of a pair of facilities is taboo for a number of iterations if they were swapped in the last iteration. However, a taboo move will be allowed if the new solution has a better objective function value than the current best solution. The tabu tenure of RTS changes between 0.9*n* and 1.1*n* dynamically during the procedure.

The advantages of RTS as compared to other adaptions of tabu search for QAP are that RTS is both efficient and robust. The robustness of RTS means that it requires less complexity and fewer parameters in its implementation. Therefore as we incorporate RTS into our BBOTS algorithm, we do not have to alter the parameter values for every benchmark instance of QAP tested.

In BBOTS algorithm, the mutation rate is now the probability that a habitat in the population will undergo RTS procedure. The number of iterations of RTS is set to *n*, which is the size of a problem. As compared to the original RTS, the number of iterations we use is relatively much lower. This is because the use of RTS in our BBOTS algorithm is limited to a quick search only. The resulting habitat after the RTS procedure will replace the original habitat if and only if the population does not contain a same habitat yet.

#### 3.2.7. BBOTS Algorithm for QAP

The BBOTS algorithm for QAP is shown in Algorithm 2.

## 4. Results and Discussion

Because of the nature of heuristic algorithms, in order to evaluate the performance of a new algorithm, it is a common practice to test it with the benchmark instances of the problem and compare its results with other existing methods. For QAP, there are benchmark instances of various sizes available from the QAPLIB. These instances are used in most of the literature. The benchmark instances of QAP can be classified into four types:

- Type I: real-life instances obtained from practical applications of QAP,
- Type II: unstructured, randomly generated instances for which the distance and flow matrices are randomly generated based on a uniform distribution,
- Type III: randomly generated instances with structure that is similar to that of real-life instances,
- Type IV: instances in which distances are based on the Manhattan distance on a grid.

The computational results are reported through 3 stages. First, we compare the results of classical BBO algorithm with mutation operator and the proposed BBOTS by applying them to selected benchmark instances from QAPLIB. After that, we further the experiments of BBOTS algorithm by testing with even more benchmark instances of QAP. Lastly, we compare the BBOTS algorithm with other state-of-the-art methods.

Both the classical BBO algorithm with mutation operator and BBOTS for QAP are programmed in MATLAB, running on the same machine with an Intel Core i3-2120 processor at 3.3 GHz. In order to fairly compare their performance, the parameter setting for both of the algorithms is the same throughout all the instances tested. The values of parameters used are indicated in Table 2.

We compare the performance of classical BBO and BBOTS algorithm for QAP by applying them on 37 benchmark instances. The computational results are shown in Table 3. The performance of the algorithms is evaluated with the following criteria:

(i) the best solution found over 10 runs,
(ii) the average deviation from the optimal or best known solution, $\bar{\delta }=100(\bar{z}-z)/z$ (%), where $\bar{z}$ is the average objective function value and *z* is the best known solution value,
(iii) the number of times (#) in which the optimal or best known solution is reached.

The benchmark instances used are of different types. Regardless of the types of instances tested, BBOTS algorithm clearly performs much better than the classical BBO with mutation operator. BBOTS algorithm is able to find the optimal or best known solutions for 36 instances out of a total of 37, while the classical BBO algorithm only manages to get 2. The average deviations from the best known solutions of BBOTS are also much lower for all the benchmark instances tested.

We further the experiments of our BBOTS algorithm by applying it to more benchmark instances of QAP.  Table 4 shows the results obtained by BBOTS algorithm for 61 benchmark instances with size ranging from 12 to 80. The parameter values used are the same as those used previously. The BBOTS algorithm is terminated at 300 iterations or when the best known solutions are found, whichever comes first.

Computational results show that the proposed BBOTS algorithm is able to find optimal or best known solutions for 57 QAP benchmark instances out of a total of 61. Most of the times, BBOTS is able to solve the instances tested on every single run. The average deviations from the best known solutions over 10 runs are at most 2.788%. Even on large instance like tai80a, the average deviation is less than 3%.

For indicative purpose, we compare the results of our BBOTS algorithm with 4 state-of-the-art QAP approaches in the literature:

(i) Iterated Tabu Search (ITS) algorithm by Misevicius (2012) [6]: the reported results are obtained using an Intel Pentium 900 MHz single-core processor;
(ii) hybrid metaheuristics combining greedy randomized adaptive search procedure and simulated annealing and tabu search (SA-TS) by Gunawan et al. (2014) [32]: the reported results are obtained using a 2.67 GHz Intel Core 2 Duo CPU;
(iii) information combination based evolutionary algorithm (ICEA) by Sun et al. (2014) [33];
(iv) genetic algorithm with a new recombination operator (GAR) by Tosun (2014) [8]: the reported results are obtained using a 2.21 GHz AMD Athlon (TM) 64 × 2 dual processor.

Since there are differences in computing hardware, termination criterion, and result reporting method, comparing the results of each algorithm is not a straightforward task. Therefore, the comparison in Table 5 should be interpreted cautiously.

The average deviations from the optimal or best known solutions are over 10 runs except for SA-TS and ICEA, which were executed for 20 and 30 times, respectively. The number of times in which the optimal or best known solutions are reached is indicated in the parentheses, if known. Should the particular authors not report their results on certain benchmark instances, the cells are left empty.

## 5. Conclusion

In this paper, we presented a biogeography-based optimization algorithm hybridized with tabu search for QAP. A classical BBO algorithm uses the mutation operator as its diversification strategy. This step often destroys the quality of habitats within the population. In the proposed BBOTS algorithm, we replaced the mutation operator with robust tabu search. The diversity among the population is maintained and, at the same time, the quality of habitats is prevented from being ruined.

The comparative results showed that BBOTS algorithm outperforms the classical BBO with mutation operator when tested with benchmark instances of QAP. The proposed BBOTS algorithm is able to obtain the optimal or best known solutions for many of the benchmark instances drawn from the QAPLIB. In particular, out of a total of 61 benchmark instances evaluated, BBOTS is able to reach the current best known solutions for 57 of them.

## Figures and Tables

**Algorithm 1:**
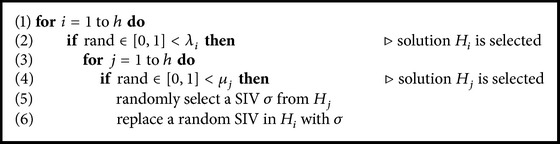
Selection strategy of migration operator.

**Algorithm 2:**
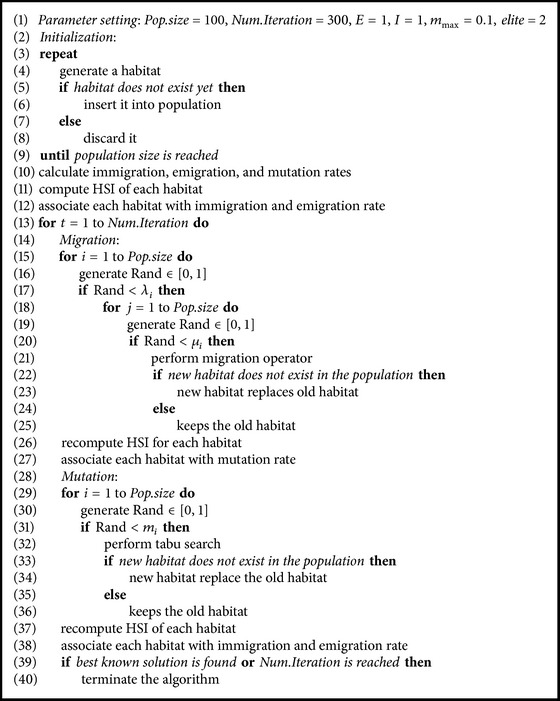
BBOTS algorithm for QAP.

**Table 1:** Comparison of characteristics for BBO and GA.

BBO	GA
Population-based	Population-based
Habitat	Chromosome
SIV	Gene
HSI	Fitness
Migration operator	Crossover operator
Mutation operator	Mutation operator

**Table 2:** Parameter setting of classical BBO and BBOTS algorithms for QAP.

Parameter	Value
Population size	100
Number of iterations	300
Maximum immigration rate	1
Maximum emigration rate	1
Maximum mutation rate	0.1
Number of elites	2

**Table 3:** Comparative results between classical BBO and BBOTS algorithms for QAP.

Instance	Best known solution	BBO			BBOTS		
		Best solution	$\overset{-}{\delta }$	#	Best solution	$\overset{-}{\delta }$	#
chr12a	9552	9988	33.654	0	9552	0.000	10
chr12b	9742	9942	28.337	0	9742	0.000	10
chr12c	11156	12336	32.854	0	11156	0.000	10
chr15a	9896	14496	97.409	0	9896	0.000	10
chr15b	7990	15298	136.683	0	7990	0.298	9
chr15c	9504	18392	112.822	0	9504	0.000	10
chr18a	11098	29870	190.483	0	11098	0.079	8
chr18b	1534	2170	51.356	0	1534	0.000	10
els19	17212548	21315378	34.293	0	17212548	0.000	10
esc16a	68	70	8.235	0	68	0.000	10
esc16b	292	292	0.000	10	292	0.000	10
esc16c	160	164	5.875	0	160	0.000	10
esc16d	16	18	22.500	0	16	0.000	10
had12	1652	1662	1.877	0	1652	0.000	10
had14	2724	2762	2.349	0	2724	0.000	10
had16	3720	3820	3.468	0	3720	0.000	10
had18	5358	5500	3.763	0	5358	0.000	10
had20	6922	7156	4.143	0	6922	0.000	10
nug12	578	590	6.574	0	578	0.000	10
nug14	1014	1086	9.310	0	1014	0.000	10
nug15	1150	1250	11.078	0	1150	0.000	10
nug16a	1610	1762	11.503	0	1610	0.000	10
nug16b	1240	1374	12.694	0	1240	0.000	10
nug17	1732	1914	12.125	0	1732	0.012	9
nug18	1930	2130	12.114	0	1930	0.000	10
nug20	2570	2868	13.416	0	2570	0.000	10
rou12	235528	247850	7.057	0	235528	0.000	10
rou15	354210	389802	12.105	0	354210	0.000	10
rou20	725522	810284	12.490	0	725522	0.062	4
scr12	31410	31410	8.243	1	31410	0.000	10
scr15	51140	58958	22.004	0	51140	0.000	10
scr20	110030	146230	37.559	0	110030	0.000	10
tai10a	135028	137362	2.974	0	135028	0.000	10
tai12a	224416	230704	8.210	0	224416	0.000	10
tai15a	388214	404108	8.843	0	388214	0.000	10
tai17a	491812	540308	11.651	0	491812	0.093	8
tai20a	703482	789348	13.658	0	705622	0.677	0

**Table 4:** Computational results of BBOTS algorithm on benchmark instances of QAP.

Instance	Best known solution	Best solution	$\overset{-}{\delta }$	#
bur26a	5426670	5426670	0.028	5
bur26b	3817852	3817852	0.000	10
bur26c	5426795	5426795	0.000	10
bur26d	3821225	3821225	0.000	10
bur26e	5386879	5386879	0.000	10
bur26f	3782044	3782044	0.000	10
bur26g	10117172	10117172	0.000	10
bur26h	7098658	7098658	0.000	10
chr12a	9552	9552	0.000	10
chr12b	9742	9742	0.000	10
chr12c	11156	11156	0.000	10
chr15a	9896	9896	0.000	10
chr15b	7990	7990	0.298	9
chr15c	9504	9504	0.000	10
chr18a	11098	11098	0.079	8
chr18b	1534	1534	0.000	10
chr20a	2192	2192	0.876	3
chr20c	14142	14142	0.604	9
els19	17212548	17212548	0.000	10
esc16a	68	68	0.000	10
esc16b	292	292	0.000	10
esc16c	160	160	0.000	10
esc16d	16	16	0.000	10
had12	1652	1652	0.000	10
had14	2724	2724	0.000	10
had16	3720	3720	0.000	10
had18	5358	5358	0.000	10
had20	6922	6922	0.000	10
kra30a	88900	88900	0.090	9
kra30b	91420	91420	0.060	6
kra32	88700	88700	0.311	7
nug12	578	578	0.000	10
nug14	1014	1014	0.000	10
nug15	1150	1150	0.000	10
nug16a	1610	1610	0.000	10
nug16b	1240	1240	0.000	10
nug17	1732	1732	0.012	9
nug18	1930	1930	0.000	10
nug20	2570	2570	0.000	10
nug21	2438	2438	0.000	10
nug22	3596	3596	0.000	10
nug24	3488	3488	0.000	10
nug25	3744	3744	0.000	10
nug27	5234	5234	0.000	10
nug28	5166	5166	0.209	4
nug30	6124	6124	0.065	2
rou12	235528	235528	0.000	10
rou15	354210	354210	0.000	10
rou20	725522	725522	0.062	4
scr12	31410	31410	0.000	10
scr15	51140	51140	0.000	10
scr20	110030	110030	0.000	10
sko42	15812	15812	0.028	9
tai10a	135028	135028	0.000	10
tai12a	224416	224416	0.000	10
tai15a	388214	388214	0.000	10
tai17a	491812	491812	0.093	8
tai20a	703482	705622	0.677	0
tai30a	1818146	1843224	1.795	0
tai80a	13499184	13841214	2.788	0
wil50	48816	48848	0.117	0

**Table 5:** Comparative results between BBOTS algorithm and state-of-the-art QAP approaches.

Instance	Best known solution	BBOTS	ITS	SA-TS	ICEA	GAR
bur26a	5426670	0.028 (5)			0.000	0.92
bur26b	3817852	**0.000 (10) **				0.65
bur26c	5426795	**0.000 (10) **				1.31
bur26d	3821225	**0.000 (10) **				0.56
bur26e	5386879	**0.000 (10) **				1.08
bur26f	3782044	**0.000 (10) **				0.56
bur26g	10117172	**0.000 (10) **				0.74
bur26h	7098658	**0.000 (10) **				
chr12a	9552	**0.000 (10) **		0.00		
chr12b	9742	**0.000 (10) **		0.00		
chr12c	11156	**0.000 (10) **		0.00		
chr15a	9896	**0.000 (10) **		0.00		
chr15b	7990	0.298 (9)		0.00		
chr15c	9504	**0.000 (10) **		0.00		
chr18a	11098	0.079 (8)		0.00		
chr18b	1534	**0.000 (10) **		0.00		
chr20a	2192	**0.876 (**3**) **		1.50		
chr20c	14142	0.604 (9)		0.00		
els19	17212548	**0.000 (10) **	** 0.00 (10) **			
esc16a	68	**0.000 (10) **				0.00
esc16b	292	**0.000 (10) **				0.00
esc16c	160	**0.000 (10) **				0.00
esc16d	16	**0.000 (10) **				0.00
had12	1652	**0.000 (10) **		0.00		0.00
had14	2724	**0.000 (10) **		0.40		0.07
had16	3720	**0.000 (10) **		0.03		0.38
had18	5358	**0.000 (10) **		0.00		0.56
had20	6922	**0.000 (10) **		0.08		1.39
kra30a	88900	0.090 (9)	0.01 (8)	0.74	0.000	
kra30b	91420	0.060 (6)	**0.00 (10) **	0.00	0.000	
kra32	88700	0.311 (7)		0.00		
nug12	578	**0.000 (10) **		0.00		
nug14	1014	**0.000 (10) **		0.00		
nug15	1150	**0.000 (10) **		0.00		
nug16a	1610	**0.000 (10) **				
nug16b	1240	**0.000 (10) **				
nug17	1732	**0.012 (9) **				
nug18	1930	**0.000 (10) **				4.97
nug20	2570	**0.000 (10) **		0.00		
nug21	2438	**0.000 (10) **		0.00		
nug22	3596	**0.000 (10) **		0.00		
nug24	3488	**0.000 (10) **		0.00		
nug25	3744	**0.000 (10) **		0.00		
nug27	5234	**0.000 (10) **		0.00		
nug28	5166	0.209 (4)		0.02		
nug30	6124	0.065 (2)	**0.00 (10) **	0.01	0.000	
rou12	235528	**0.000 (10) **		0.00		
rou15	354210	**0.000 (10) **		0.00		
rou20	725522	0.062 (4)		0.03		
scr12	31410	**0.000 (10) **		0.00		
scr15	51140	**0.000 (10) **		0.00		
scr20	110030	**0.000 (10) **		0.00		
sko42	15812	0.028 (9)	**0.00 (10) **	0.14	0.000	
tai10a	135028	**0.000 (10) **		0.00		
tai12a	224416	**0.000 (10) **		0.00		
tai15a	388214	**0.000 (10) **		0.00		
tai17a	491812	0.093 (8)		0.00		
tai20a	703482	0.677 (0)	**0.00 (10) **	0.16	0.168	
tai30a	1818146	1.795 (0)	**0.00 (10) **	1.51	0.276	
tai80a	13499184	2.788 (0)	**0.36 (1) **	3.57	0.998	10.94
wil50	48816	0.117 (0)	**0.06 (4)**	0.11		7.18
